# Impact of adjuvant chemotherapy on patients with ypT0–2 ypN0 rectal cancer after neoadjuvant chemoradiation: a cohort study from a tertiary referral hospital

**DOI:** 10.1186/s12957-018-1455-x

**Published:** 2018-08-02

**Authors:** Christian Galata, Kirsten Merx, Sabine Mai, Timo Gaiser, Frederik Wenz, Stefan Post, Peter Kienle, Ralf-Dieter Hofheinz, Karoline Horisberger

**Affiliations:** 1 Department of Surgery, University Hospital Mannheim, Medical Faculty Mannheim, University of Heidelberg, Theodor-Kutzer-Ufer 1-3, 68167 Mannheim, Germany; 2 Interdisciplinary Tumor Centre, III. Department of Internal Medicine, University Hospital Mannheim, Medical Faculty Mannheim, University of Heidelberg, Mannheim, Germany; 3 Institute for Radiotherapy and Radiooncology, University Hospital Mannheim, Medical Faculty Mannheim, University of Heidelberg, Mannheim, Germany; 4 Institute for Pathology, University Hospital Mannheim, Medical Faculty Mannheim, University of Heidelberg, Mannheim, Germany; 5 Department of Visceral and Transplant Surgery, Universitätsspital Zürich, Zürich, Switzerland; 6 Department of Surgery, Theresienkrankenhaus Mannheim, Mannheim, Germany

**Keywords:** Rectal neoplasms, Neoadjuvant therapy, Chemoradiotherapy, Adjuvant chemotherapy, Disease-free survival

## Abstract

**Background:**

To investigate the importance of adjuvant chemotherapy in locally advanced rectal cancer (≥ cT3 or N+) staged ypT0–2 ypN0 on final histological work-up after neoadjuvant chemoradiation and radical resection.

**Methods:**

The clinical course of patients with rectal cancer and ypT0–2 ypN0 stages after neoadjuvant chemoradiation and radical resection was analyzed from 1999 to 2012. Patients were divided into two groups depending on whether adjuvant chemotherapy was administered or not. Overall survival, distant metastases, and local recurrence were compared between both groups.

**Results:**

Fifty-four patients with adjuvant (ACT) and 50 patients without adjuvant chemotherapy (NACT) after neoadjuvant chemoradiation followed by radical resection for rectal cancer were included in the analysis. Mean follow-up was 68 ± 33.7 months. One patient without adjuvant chemotherapy and none in the ACT group developed a local recurrence. Five patients in the NACT group and three patients in the ACT group had distant recurrences. Median disease-free survival for all patients was 65.5 ± 34.5 months. Multivariate analysis showed adjuvant chemotherapy to be the most relevant factor for disease-free and overall survival. Patients staged ypT2 ypN0 showed a significantly better disease-free survival after application of adjuvant chemotherapy. Disease-free survival in ypT0–1 ypN0 patients showed no correlation to the administration of adjuvant chemotherapy.

**Conclusion:**

Administration of adjuvant chemotherapy after neoadjuvant chemoradiation and radical resection in rectal cancer improved disease-free and overall survival of patients with ypT0–2 ypN0 tumor stages in our study. In particular, ypT2 ypN0 patients seem to profit from adjuvant treatment.

## Background

Neoadjuvant chemoradiation is a considered standard treatment for locally advanced rectal cancer [1]. Current guidelines for the treatment of colorectal cancer in Germany recommend the administration of adjuvant chemotherapy for all rectal cancer patients after neoadjuvant chemoradiation and total mesorectal excision (TME), regardless of the postoperative pathologic staging result [2]. This recommendation is based on the CAO/ARO/AIO-94 and FFCD 9203 studies [1, 3]. However, hard evidence is lacking, especially for patients staged ypT0–2 ypN0. While an exploratory analysis suggested that particular patients with good response (ypT0–2) benefit from adjuvant chemotherapy [4] randomized controlled trials addressing the same question showed no benefit for adjuvant chemotherapy [5, 6]. However, these trials have relevant methodological restrictions. While a recent pooled analysis showed positive effects for adjuvant chemotherapy, another recent meta-analysis failed to do so [7, 8].

In adherence to the German national guidelines from before 2008, ypT0–2 ypN0 patients were then not treated with postoperative chemotherapy (NACT) at our institution. After the introduction of the amended guidelines in 2008, adjuvant treatment was routinely administered to the same group of patients (ACT). In the present study, we investigated patients with locally advanced rectal cancer in clinical staging (UICC stages II and III) treated with neoadjuvant chemoradiation and TME and then staged ypT0–2 ypN0. On the basis of a prospectively maintained database, the oncologic outcomes of these patients were analyzed.

## Methods

### Ethics approval

The institutional review board reviewed and approved the protocol; the study was conducted in accordance with the Declaration of Helsinki.

### Patient selection

All surgically treated colorectal carcinomas at the Department of Surgery, University Hospital Mannheim, Germany, between 1999 and 2012 were retrospectively analyzed on the basis of prospective databases. Patients with locally advanced rectal cancer who underwent neoadjuvant chemoradiation and subsequent TME in curative intent were eligible for the study when diagnosed ypT0–2 ypN0 in postoperative pathological staging. Exclusion criteria were postoperative death (in-hospital mortality), UICC stage IV and recurrent disease, or missing information on whether adjuvant chemotherapy was administered. Primary outcome measure was disease-free survival (DFS). Disease was defined as the event of local and/or distant recurrence during follow-up. DFS was defined as absence of local and/or distant recurrence and death by any cause during follow-up after primary hospital stay.

### Pre-treatment evaluation

The presence of adenocarcinoma was confirmed by pathological examination in all cases. Clinical staging was performed using rigid rectoscopy, endorectal ultrasound, radiographic imaging of the chest, and abdominal ultrasound. Routine performance of magnetic resonance imaging (MRI) of the pelvis was introduced in 2003. Computed tomography (CT) scans of the thorax and/or abdomen were obtained in the majority of cases.

### Preoperative chemoradiation and surgery

Neoadjuvant chemoradiation was administered when locally advanced rectal cancer was diagnosed (uT3-4, uN+). As preoperative chemotherapy regimen, capecitabine, capecitabine + irinotecan (XELIRI), XELIRI + cetuximab, capecitabine + oxaliplatin (XELOX), intravenous 5-FU, or panitumumab were used. Radiation therapy was applied as external-beam radiation with a target dose of 50.4 Gy. TME was scheduled 4 to 5 weeks after completing neoadjuvant chemoradiation before 2008, and 8 to 12 weeks after completion of chemoradiation for patients from 2008 till 2012.

### Pathology investigation

Resected specimens were fixated in formalin and pathological work-up was done according to published standards [9]. If no residual tumor was apparent, the initial tumor-bearing area was sliced and embedded. Tumor regression grade was determined based on the classification proposed by the Japanese Society for Cancer of the Colon and Rectum (JSCCR) [10].

### Postoperative chemotherapy

According to national colorectal cancer guidelines before the year 2008, patients were not offered postoperative chemotherapy when diagnosed ypT0–2 ypN0 in final pathological staging. After 2008, adjuvant chemotherapy became the treatment of choice for those patients when no contraindications were present. For adjuvant chemotherapy capecitabine, XELOX or intravenous 5-FU was used.

### Statistical analysis

Baseline characteristics of all patients together were evaluated with respect to their influence on outcome. The characteristics were then compared between patients who received adjuvant chemotherapy and those who did not. Comparisons of frequencies between the two groups were performed using the Student’s *t* test or the chi-square test. Differences of non-parametric quantitative data were analyzed using the Mann-Whitney *U* test. Kaplan-Meier estimates were computed for recurrence and survival and were compared between the two treatment groups using the log-rank test. A *p* value < 0.05 was considered statistically significant. All calculations were made with the SPSS version 22.0 (IBM© SPSS® Statistics).

## Results

### Patients’ characteristics

Initial screening of the database returned 131 patients staged ypT0–2 ypN0 rectal cancer between 1999 and 2012 out of 397 patients who had received neoadjuvant treatment. Twenty-seven patients were excluded due to the above mentioned exclusion criteria. A total of 104 patients met the inclusion criteria, 28 females (26.9%) and 76 males (73.1%) with a mean age of 62.0 ± 10.7 years. Low rectal cancer was present in 48 patients (46.2%); 52 patients (50%) had cancers of the mid rectum, and 4 patients (3.8%) of the upper rectum. Median dose of delivered radiation was 50.4 Gy (range 36 to 50.4 Gy). Sphincter-preserving operation was performed in 79.8% of the patients (*n* = 83). Postoperative chemotherapy was given to 54 patients (51.9%), while 50 patients (48.1%) did not receive adjuvant treatment. Ten of the patients (18.5%) who had received adjuvant therapy had surgery before 2008. In the group without adjuvant therapy, 41 patients (82%) were operated before 2008.

Data on gender, age, tumor height, preoperative radiation dose, and type of operation did not differ significantly between ACT and NACT (Table 1). A total of 46 patients (44.2%) were diagnosed ypT0 or ypT1 whereas 58 patients (55.8%) were diagnosed ypT2 in the final pathological examination. Distribution of ypT0, T1, and T2 showed a non-significant trend towards a higher rate of ypT0 in the patient group that received no adjuvant treatment (Table 1). Patients with ypT0–1 versus ypT2 showed no difference between the treatment groups. Neither the distribution of tumor regression grading nor the number of retrieved lymph nodes showed differences between both groups.

**Table 1:** Patient characteristics of patients without and with adjuvant chemotherapy. In one patient, regression grade could not be determined

	No adjuvant therapy (*n* = 50)	Adjuvant therapy (*n* = 54)	*p* value
Age	62.9 ± 11.6	61.2 ± 9.8	0.414
Sex (female/male)	36/14	40/14	0.829
Abdominoperineal resection	10/50	11/54	1.0
Anastomotic leakage	9/40	3/43	0.062
T stage			
ypT0	20 (40%)	12 (22%)	0.064
ypT1	6 (12%)	8 (15%)	
ypT2	24 (48%)	34 (63%)	
T stage			
ypT0–1	26	20	0.167
ypT2	24	34	
Lymph nodes retrieved	13.1 ± 0.7	13.5 ± 0.7	0.687
Lymph nodes			
< 12	12 (24%)	12 (22.2%)	1.0
≥ 12	38 (76%)	42(77.8%)	
Regression grade (JSCCR)			
TRG 0	0	1 (2%)	0.384
TRG 1	11 (22%)	9 (17%)	
TRG 2	20 (49%)	32 (60%)	
TRG 3 (pCR)	18 (36%)	12 (22%)	

### Oncologic outcomes according to adjuvant chemotherapy

Mean follow-up was 68.0 months (± 33.7) for all patients eligible for the study. Follow-up time was significantly longer in the group without adjuvant chemotherapy (*p* < 0.005; Table 2). Mean disease-free survival was 65.5 ± 34.5 months. Overall recurrence of the disease was seen in nine patients (8.7%). Metachronous metastasis occurred in eight cases (7.7%) and locoregional recurrence in one patient (0.96%).

**Table 2:** Follow-up, local, and distant recurrence in patients without and with adjuvant chemotherapy

	No adjuvant therapy (*n* = 50)	Adjuvant therapy (*n* = 54)	*p* value
Follow-up (months)	82.2 ± 38.7	54.7 ± 21.4	0.003
Local recurrence	1	0	0.481
Distant recurrence	5	3	0.477

**Fig. 1:**
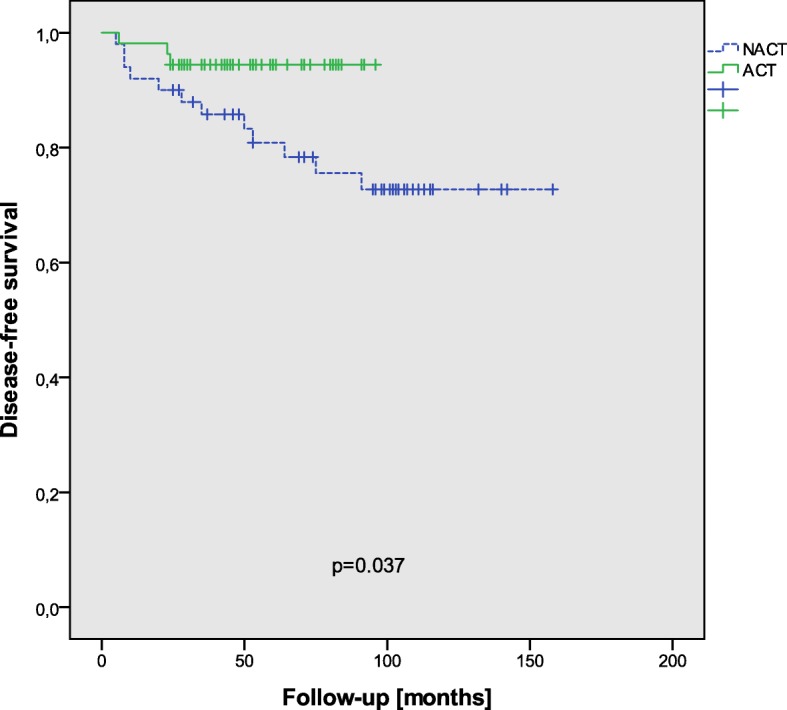
DFS in all patients with respect to adjuvant chemotherapy

In the univariate analysis, age, sex, tumor height, and extirpation had no influence on disease-free survival. Log-rank tests showed that adjuvant chemotherapy had no influence on local recurrence (*p* = 0.382), distant metastasis (*p* = 0.54), or overall recurrence (*p* = 0.382) but on disease-free survival (*p* = 0.037) and overall survival (*p* = 0.017) (Fig. 1). The 3-year OS and DFS were 98 and 94% in the ACT group, respectively, and 87 and 86% in the NACT group.

Anastomotic leakage showed a statistical trend towards influencing overall (*p* = 0.053) but not disease-free survival (*p* = 0.435). Adjuvant chemotherapy, after stratification for anastomotic leakage, demonstrated a statistically significant effect on disease-free survival in patients without leakage (*p* = 0.016); however, there was no significant influence of adjuvant chemotherapy on disease-free survival in patients with anastomotic leakage (*p* = 0.293).

**Fig. 2:**
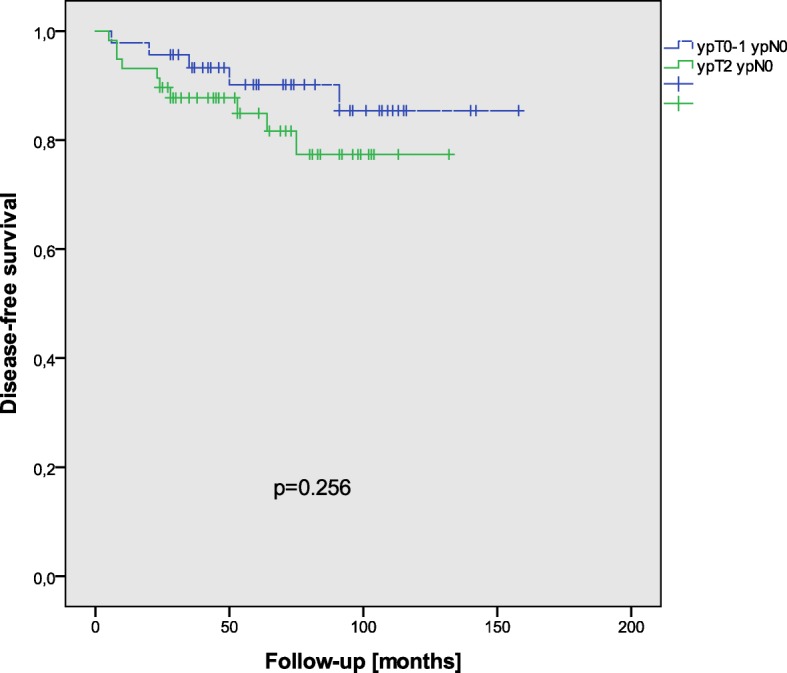
DFS in all patients with respect to T-stage groups (ypT0–1 ypN0 versus ypT2 ypN0)

**Fig. 3:**
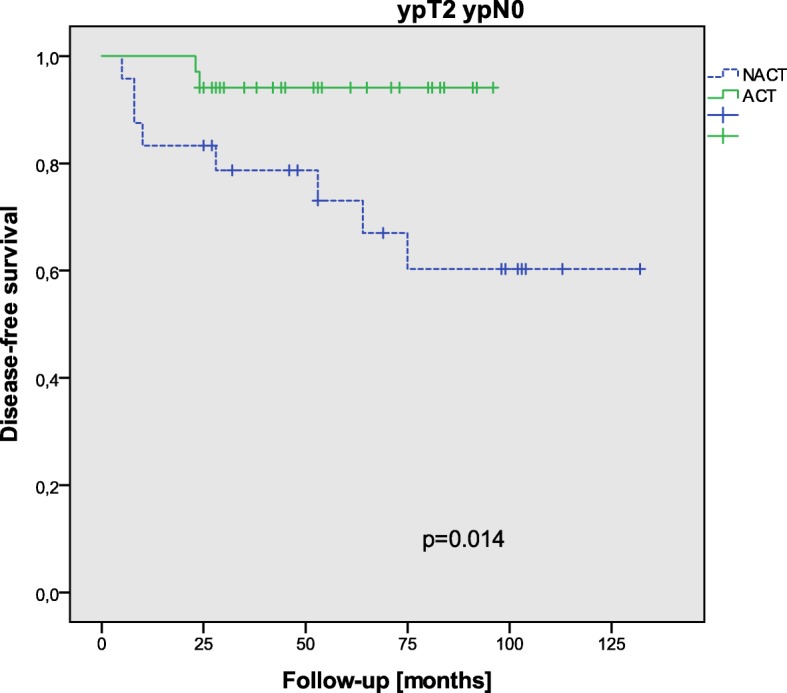
DFS in ypT2 ypN0 patients with respect to adjuvant chemotherapy

ypT stages did not influence disease-free survival (*p* = 0.513), and also ypT stage groups (ypT0–1 versus ypT2) were not correlated to disease-free survival (*p* = 0.265) (Fig. 2). After stratification along these groups, no significant correlation with adjuvant chemotherapy could be seen concerning disease-free survival in ypT0–1 (*p* = 0.556); however, ypT2 patients showed a significantly better disease-free survival after adjuvant chemotherapy (*p* = 0.014) (Fig. 3). In ypT0 (*p* = 0.195) and ypT1 (*p* = 0.386), no correlation between adjuvant chemotherapy and disease-free survival could be detected. After stratification in groups of pCR (pathological complete response) versus ypT1–2, disease-free survival showed no significant correlation to adjuvant treatment in ypT0 patients (*p* = 0.195), and only marginally in ypT1–2 patients (*p* = 0.056). The 3-year DFS and OS in the ACT group were both 100% in ypT0, 100 and 88% in ypT1, and 94 and 100% in ypT2, respectively, and in the NACT group both 90% in ypT0, both 100% in ypT1, and 79 and 82% in ypT2.

When patients were classified in groups with more or less than 12 lymph nodes harvested, the number of lymph nodes harvested did not influence the disease-free survival by itself (*p* = 0.821). The interaction of lymph nodes harvested and adjuvant chemotherapy showed a significantly better disease-free survival in patients with more than 12 lymph nodes (*p* = 0.009) but no significant influence of adjuvant chemotherapy in patients with less than 12 lymph nodes (*p* = 0.809).

Of the patients, 90% had at least half of the indicated chemotherapy cycles, 83% had 5 or 6 chemotherapy cycles, and 7% had 3 or 4 cycles (Table 3). Completeness of chemotherapy had no influence on the outcome.

**Table 3:** Completion of adjuvant chemotherapy (in three patients, the number of cycles could not be clarified anymore)

Completeness of chemotherapy	5–6 cycles	3–4 cycles	1–2 cycles
ACT group (*n* = 54)	45 (83%)	4 (7%)	2 (4%)

## Discussion

Introduction of neoadjuvant chemoradiation for rectal adenocarcinoma has in combination with TME surgery led to reduce rates of locoregional recurrence [1]. This improvement of local control, however, did not result in prolonged overall survival [11]. Our data show a significant benefit from adjuvant treatment for disease-free and overall survival but no benefits with respect to recurrence.

Adjuvant chemotherapy after neoadjuvant treatment and TME surgery is administered with the intention of reducing the incidence of distant metastasis and thereby improving survival. Although this has been prospectively investigated in several trials, controversy remains [5, 6]. In the just recently published study by Breugom et al., patients with ypTNM stage 0 or I were explicitly excluded which was also criticized [8, 12, 13]. A meta-analysis identified this subgroup to profit the most from adjuvant chemotherapy [14]. Maas et al. found the most pronounced effect of adjuvant chemotherapy on disease-free survival in ypT1–2 patients both in comparison to higher stages but also to pCR patients [7]. Our analysis found the most pronounced effect of adjuvant chemotherapy in ypT2 patients. The theoretical consideration that tumors with the combination of responsiveness (shown by downstaging) and continued considerable risk for local and distant recurrence (> ypT1) would profit from adjuvant treatment might in particular hold true for ypT2 [4].

Another restriction in the analysis of Breugom et al. is that the majority of the patients received bolus 5-FU [8]. However, an explanatory phase III trial showed better disease-free survival after perioperative treatment with capecitabine than with 5-FU [15]. In our analysis, only two patients received 5-FU postoperatively; therefore, a comparison of the effect of the two agents cannot be undertaken.

Our results are in conflict with the EORTC 22921 study that previously reported ypT1–2 patients to benefit from postoperative chemotherapy after 5 years; however, recently published late results after 10 years showed no improvement in disease-free or overall survival [4, 16]. Meta-analyses presented inherently contradictory results with respect to the positive effects of adjuvant chemotherapy on disease-free and overall survival [7, 8, 17, 18]. However, it is difficult to draw conclusions from these meta-analyses as the included studies have relevant shortcomings. As mentioned above, TME was not mandatory in some of the trials; others revealed a questionable quality of surgery with a R1 rate above 10% and finally, but most important, many of the studies showed a high percentage of patients not undergoing any adjuvant chemotherapy or not the initially planned number of cycles [5]. Low adherence to planned postoperative chemotherapy is one of the major problems of the available randomized trials, and it is a serious problem for interpretation of non-significant results as a proof for ineffectiveness of adjuvant chemotherapy [6, 8, 19]. In the EORTC 22921 trial, only 41% of the patients received complete chemotherapy [13, 16].

Anastomotic leakage is a major problem for patients who actually would have been eligible for adjuvant therapy. In our cohort, anastomotic leakage showed a trend towards negatively influencing application of adjuvant chemotherapy (*p* = 0.062). This is well in accord with general clinical experience that anastomotic leakage often prevents application of chemotherapy.

The results of the PROCTOR-SCRIPT trial challenge our study, as this is the first randomized trial on the application of adjuvant chemotherapy in neoadjuvantly treated patients with rectal cancer [6]. In this study, no benefit from adjuvant chemotherapy could be detected. However, again, there are limitations in this study. The trial had to be closed earlier due to poor patient recruitment and survival was better than expected suggesting that the trial was probably underpowered. In the PROCTOR part of the trial, only 50% of the patients had a CT or MRI scan before treatment, so inaccuracy of staging is probably a major bias. Patients were preoperatively treated either with 5 × 5 Gy or with long-term chemoradiation; however, the longstanding oncological results of a randomized comparison of these two therapy schedules are still awaited [20]. Furthermore, stagewise analysis was not performed in the PROCTOR-SCRIPT trial and the number of retrieved lymph nodes not reported [6].

The question if the number of lymph nodes retrieved during surgery would influence long-term outcome respectively the application of chemotherapy and thereby the outcome has to be addressed. Most guidelines recommend investigation of at least 12 lymph nodes for determining final pathologic tumor stage [21]. In several studies, the number of detected locoregional lymph nodes was decreased after neoadjuvant chemoradiation [22, 23]. However, when < 12 lymph nodes are investigated, metastasis could be missed and histopathological stage underestimated. As performance of adjuvant treatment is stage-dependent also patients that would need therapy are then excluded. When intensified pathology work-up of the specimens is performed and more lymph nodes are evaluated, the number of metastatic lymph nodes may raise thereby possibly resulting in stage migration (“Will Rogers phenomenon”) [24]. While some studies indicate an association between the number of harvested lymph nodes and oncologic outcome [24], the same authors could not reproduce these results when neoadjuvant chemoradiation was administered [25]. Recent studies on this topic continue to give conflicting results; therefore, the significance of retrieving more than 12 lymph nodes remains unclear [26, 27]. In the present study, retrieval of less than 12 lymph nodes showed no influence of adjuvant chemotherapy with respect to disease-free survival, while more than 12 lymph nodes and adjuvant chemotherapy were correlated to a better disease-free survival.

There are several limitations to our study. First, this is a retrospective study. Patients were not prospectively randomized and selection bias cannot be excluded, even though the groups were well matched in size, age, gender, and tumor-specific parameters. As the indication for adjuvant therapy changed in 2008 in Germany, the comparison could be described as historical. Second, follow-up time was significantly longer in the NACT group. The difference is explainable by the consecutive change of guidelines. These two points in turn can be regarded as strength of this study, making it a “quasi-RCT.” Furthermore, follow-up in patients who received adjuvant chemotherapy still was 54 months in mean.

Third, a possible sign of selection bias is the higher proportion of ypT0 patients in the group without adjuvant chemotherapy. In fact, clinicians are often averse to the application of adjuvant chemotherapy in pCR patients. Breugom et al. criticized the retrospective character of the study by Maas et al. and the fact that the other study supporting adjuvant chemotherapy was a meta-analysis [7, 14, 28]. However, a meta-analysis usually reduces the risk of confounding.

A more detailed analysis of surgical complications other than anastomotic leakage could not be performed. Even if the database was prospectively performed and updated, the number of parameters documenter increased over time, e.g., the Clavien-Dindo complication grading was only introduced at a later stage. However, anastomotic leakage, which was adequately documented in the database, is one of the most severe complications in rectal surgery and most often the reason why adjuvant chemotherapy is delayed or not started at all. Moreover, leakage has been shown to influence the oncological outcome, and as both groups demonstrated a comparable leakage rate, this factor can be ruled out as a biasing factor.

At last, the sample size is too small to be able to evaluate statistical significant difference in rare incidences such as local recurrence that occurred only once.

Regardless of these limitations, the results support current guideline recommendations that in patients with ypT0–2 tumors adjuvant chemotherapy should continue to be administered, especially in ypT2 stages.

## Conclusion

Administration of adjuvant chemotherapy after neoadjuvant chemoradiation and radical resection in rectal cancer improved disease-free and overall survival of patients with ypT0–2 ypN0 tumor stages in our study. In particular, ypT2 ypN0 patients seem to profit from adjuvant treatment.
